# Leveraging Electronic Health Records to Learn Progression Path for Severe Maternal Morbidity

**DOI:** 10.3233/SHTI190201

**Published:** 2019-08-21

**Authors:** Cheng Gao, Sarah Osmundson, Xiaowei Yan, Digna Velez Edwards, Bradley A. Malin, You Chen

**Affiliations:** a Department of Biomedical Informatics, Vanderbilt University Medical Center, Nashville, TN, United States; b Department of Obstetrics and Gynecology, Vanderbilt University Medical Center, Nashville, TN, United States; c Sutter Research, Development and Dissemination, Sacramento, CA, United States; d Department of Biostatistics, Vanderbilt University Medical Center, Nashville, TN, United States; e Department of Electrical Engineering and Computer Science, Vanderbilt University, Nashville, TN, United States

**Keywords:** Electronic health records, Pregnancy Risk Factors

## Abstract

Severe maternal morbidity (SMM) encompasses a wide range of serious health complications that would likely result in death without in-time medical attention. It has been recognized that various demographic factors (e.g., age and race) and medical conditions (e.g., preeclampsia and organ failure) are associated with SMM. However, how medical conditions develop into SMM is seldom investigated. We hypothesize that SMM has a progression path, which is associated with a sequence of risk factors rather than a set of independent individual factors. We implemented a data-driven framework that leverages electronic health records (EHRs) in the antepartum period to learn the temporal patterns and measure their relationships with SMM during the delivery hospitalization. We evaluate the framework with two years of data from 6,184 women who had delivery hospitalizations at Vanderbilt University Medical Center. We discovered 69 temporal patterns, 12 of which were confirmed to be significantly associated with SMM.

## Introduction

Severe maternal morbidity (SMM) such as severe hemorrhage, uterine rupture, organ failure, embolism, pulmonary embolism, and seizure is a physical condition that either result from or is aggravated by pregnancy and has an adverse effect on a woman’s health.[1] It has been recognized that women with SMM often have a longer length of stay, higher medical cost at the time of delivery, and potentially worse neonatal and postpartum outcomes than those without SMM [2]. SMM has been utilized as an indicator of the quality of maternal health and the cost of healthcare.[3; 4]

Although the definitions of SMM are different from one study to another, its prevalence has steadily increased worldwide. [4; 5] Around 60,000 women are now affected in the US every year. [6; 7] Learning the risk factors of SMM, and predicting it early can potentially improve the quality of women’s health and reduce healthcare costs.

Given the complexity of SMM and its worldwide prevalence, health practitioners and researchers at the national and state level have made considerable progress to learn the risk factors for SMM [9; 10]. It has been recognized that various factors including demographics (e.g., age and race), lifestyle (e.g., tobacco use and alcohol consumption), and medical conditions (e.g., preeclampsia and eclampsia, gestational diabetes mellitus, and prior cesarean section) are related to SMM. Yet almost all studies to date relied on hypothesis-driven approaches to learn risk factors, which have several drawbacks. First, they only investigated a small number of predetermined risk factors and neglected many other factors which may contribute to SMM. This is due to the fact that these studies relied on experts’ experiences to determine candidate risk factors which can cost a huge amount of manual effort. Second, most studies did not consider the temporal relationships between risk factors. This is important because women with SMM, rarely transition directly from a healthy state to SMM or death. Rather they tend to progress along a continuum from one health status to another and, eventually, to severe morbidity or death.

To overcome these drawbacks, we introduced a data-driven framework to learn the progression path for SMM in the antepartum period (i.e., pregnancy before delivery). We focused on this period because our aim was to discover temporal patterns of risk as early as possible so that appropriate interventions could be applied to prevent SMM or reduce harms caused by SMM. In addition, this framework is built using on-the-shelf data and, thus, considered a large number of candidate risk factors with minimal manual effort. In particular, we leveraged data in electronic health records (EHRs) to learn temporal patterns between maternal conditions, in terms of International Classification of Disease (ICD) codes and measure the relationships of the temporal patterns with SMM. This work is notable because it shifts the investigations of SMM risk factors from atemporal to temporal manner.

## Methods

**Figure 1.**
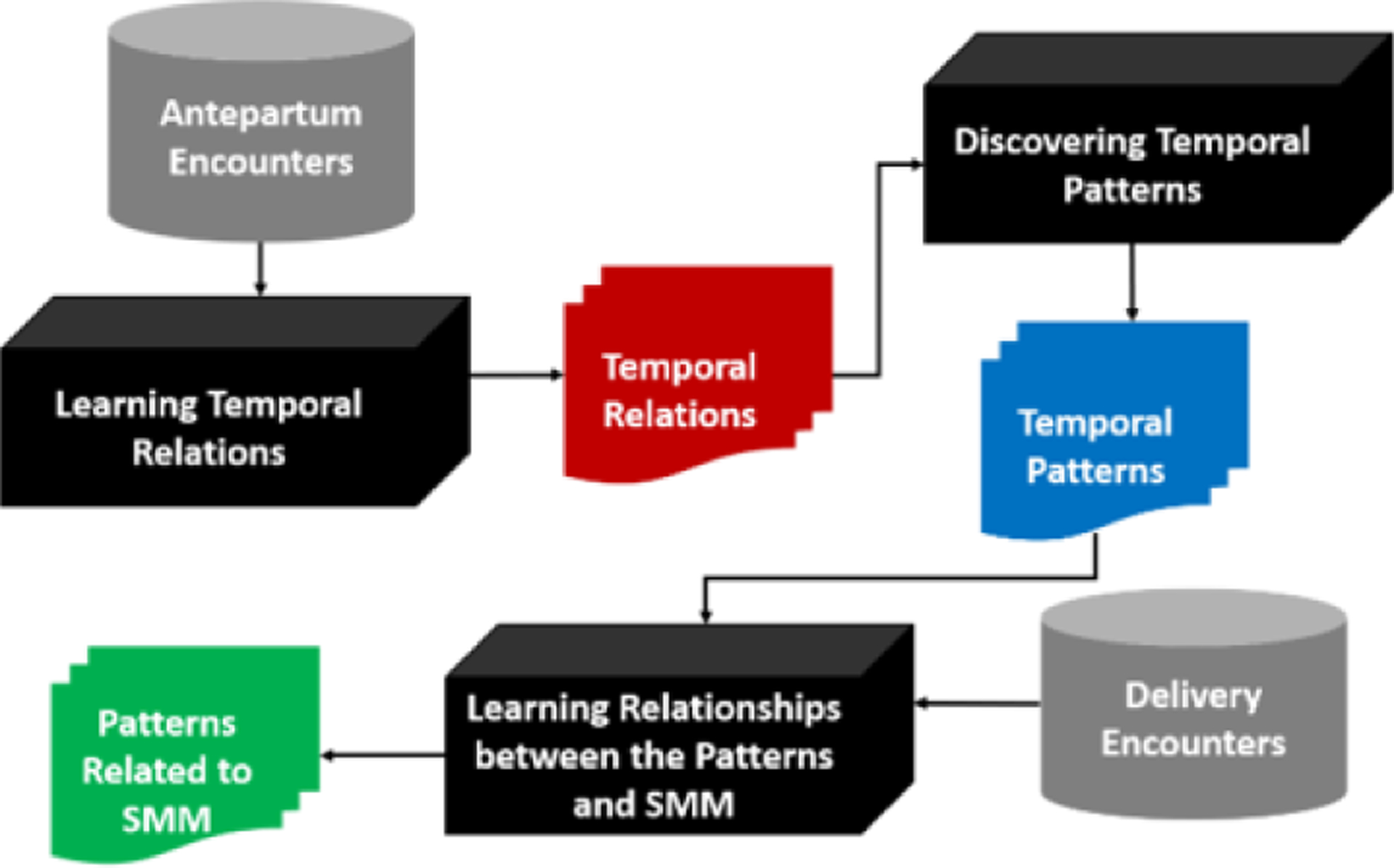
An overview of the data-driven framework to learn temporal patterns related to SMM.

Our data-driven framework consists of three components: i) a data mining algorithm to learn temporal relations between diagnoses in antepartum period, ii) a statistical model to identify temporal patterns from temporal relations, and iii) a regression model to identify SMM related temporal patterns. An overview of the framework is depicted in Figure 1.

### Dataset

The data is based on 6,184 obstetric patients who had a delivery encounter at Vanderbilt University Medical Center (VUMC) between 2015 to 2017. For each patient, the data documents: 1) demographics (age and race), and 2) clinical concepts during antepartum and delivery encounters. For each encounter, we have admission and discharge dates and the set of ICD-9 codes. The data in the antepartum encounters were leveraged to learn temporal relations between ICD-9 codes and the data in the delivery encounters were used to determine if a patient experienced SMM or not. We used 25 SMM indicators in the form of ICD-9 codes, as defined by the Centers for Disease Control and Prevention (CDC) to identify SMM cases [1]. Summary statistics of patients with and without SMM are shown in Table 1.

#### Encounter:

An encounter corresponds to a hospital visit, which has the admission and discharge date. Encounters in this paper are denoted as *E*_1_, *E*_2_, ⋯ , *E*_*n*_.

#### Code:

A diagnosis is specified as an ICD-9 code or procedure code. ICD-9 codes are used for billing services and are assigned to EHRs by physicians in patient care or by medical coders after the discharge of an encounter. The time when an ICD code is assigned to EHRs of a patient within an encounter does not necessarily represent the exact time when the patient received the corresponding diagnosis. However, ICD-9 codes across encounters should have temporal relations. For instance, if code 648.01 was assigned in one encounter ranging from February 3 to February 4, and code 655.83 was assigned in another encounter ranging from March 3 to March 5, then 655.83 was diagnosed after 648.01, and the temporal relationship exists between them. We denoted a code as *c*_*i*_. If an encounter *E*_*k*_ has *m* different codes, then we will represented all codes in the *E*_*k*_ as {*c*_1_, *c*_2_, ⋯ , *c*_*m*_}.

#### Code sequence:

**Figure 2.**
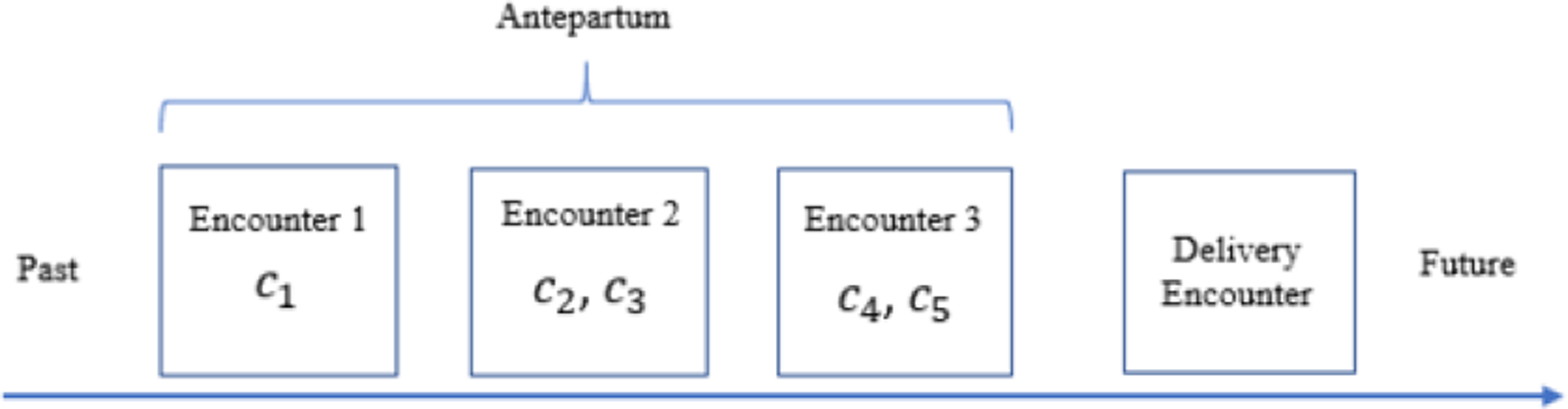
An example of four temporal perinatal encounters of a patient. Codes were assigned in each of the three antepartum encounters.

A code sequence is an ordered series of codes coming from disparate encounters. For example, as shown in Figure 2, this patient has three antepartum encounters with 4 code sequences: {*c*_1_ → *c*_2_ → *c*_4_, *c*_1_ → *c*_2_ → *c*_5_, *c*_1_ → *c*_3_ → *c*_4_, *c*_1_ → *c*_3_ → *c*_5_}.

### Building Temporal Relations

We designed a sliding window-based algorithm to build temporal relations between diagnosis codes. The ordered relation between a pair of codes within a code sequence as:

(1) $$Code_{\text{relation}}\left(c_{i},\;c_{j}\right)\;=\;\{\begin{array}{cc} \frac{1}{{\left(p\left(c_{i}\right)-\;p\left(c_{j}\right)\right)}^{2}}, & (0\;<\;p\left(c_{j}\right)-p\left(c_{i}\right)\;\leq \alpha \\ 0, & \text{otherwise} \end{array}$$

where *p*(*c*_*i*_) is the position of a code *c*_*i*_ in a code sequence and *α* is the window size. The position of the first code of a sequence is set as 1 and the position of the last code is the length of a sequence.

**Figure 3.**
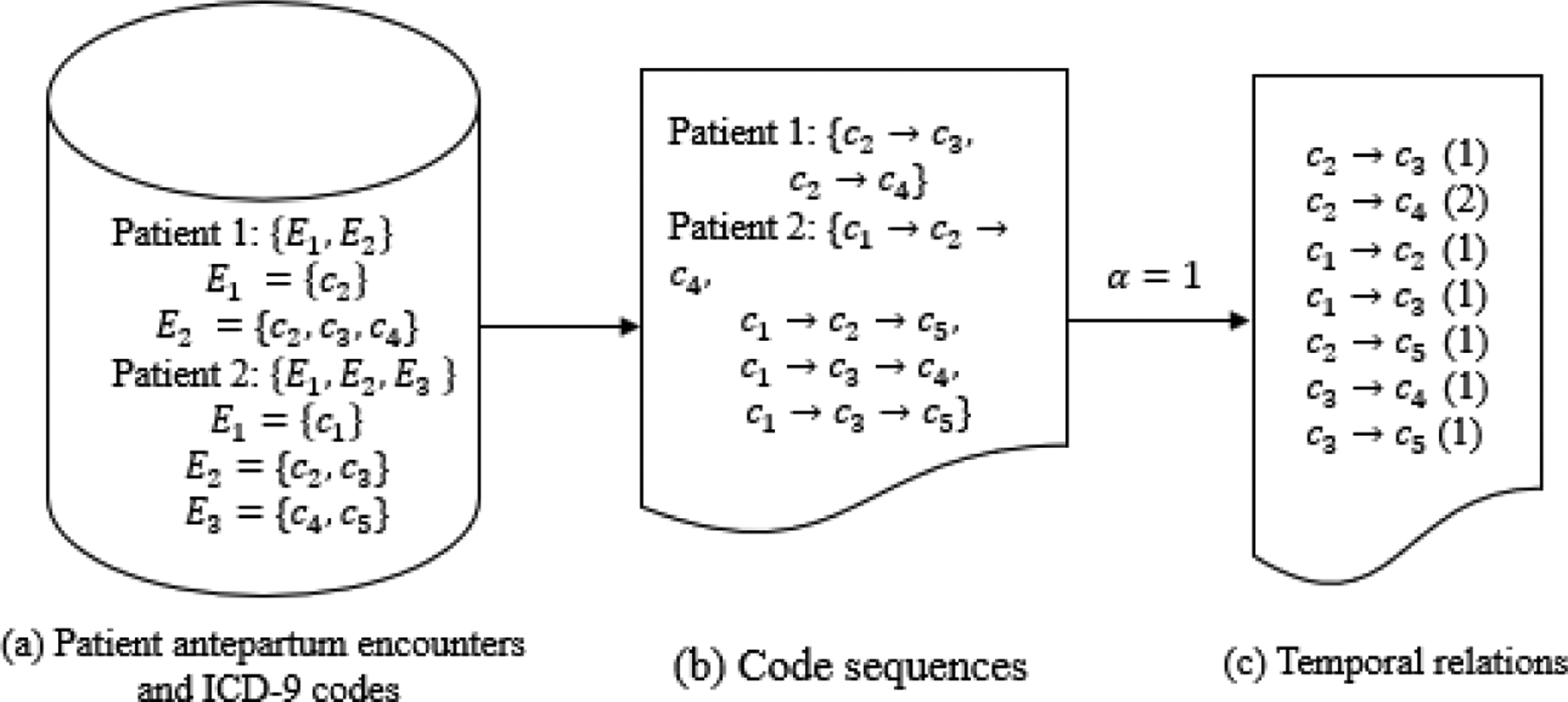
An example of learning temporal relations from six code sequences extracted from five encounters

Figure 3 shows an example to illustrate the algorithm. There are two patients P1 and P2, with 2 and 3 encounters, respectively. Each encounter has several diagnosis codes assigned. For the first patient, the first encounter contains one code *c*_2_ and the second encounter contains three codes *c*_2_, *c*_3_, *c*_4_ . The code sequences for the first patient are {*c*_2_ → *c*_3_, *c*_2_ → *c*_4_}. It is notable that *c*_2_ → *c*_2_ is not included in code sequences because *c*_2_ appears in both encounters and *c*_2_ → *c*_2_ does not convey any temporal information between encounters. The code sequences for the second patient are {*c*_1_ → *c*_2_ → *c*_4_, *c*_1_ → *c*_2_ → *c*_5_, *c*_1_ → *c*_3_ → *c*_4_, *c*_1_ → *c*_3_ → *c*_5_}. If we set window size *α* is 1, we can learn the strength of temporal relation for each pair of codes within a code sequence via (Eq. 1). The total strength of the temporal relation for a pair of codes is the summary of temporal strength of that pair of codes across all sequences. Temporal relations and their strengths are depicted in Figure 3c For instance *c*_2_ → *c*_4_ appears in two code sequences and the its temporal strength in each of the code sequence is 1 accoridng to (Eq. 1), and thus its total strength is accumulated across the two sequences.

### Discover temporal patterns

We applied statistical matching methods [12] along with statistical models to identify temporal patterns from the learned temporal relations. For an investigated temporal relation *c*_*i*_ → *c*_*j*_, we defined patients containing *c*_*i*_ as cases, and patients without containing *c*_*i*_ as controls. We selected most similar controls and cases in terms of age, race and EHR length. Based on the selected cases and controls, we used a Chi-square test to test the significance of *c*_*i*_ → *c*_*j*_ after Bonferroni correction. A code relationship was considered a temporal pattern when the *p* value was smaller than 0.05.

### Learn relationships of temporal pattern with SMM

We used logistic regression to model relationships between antepartum temporal patterns and SMM during delivery hospitalization. Age, race, and length of EHR are included in the model as confounders. For each temporal pattern, logistic regression is applied to evaluate its adjusted relative risk (aRR) on SMM. The significant temporal patterns are reported with the 95% confidence interval of aRR.

## Result

### Temporal relation patterns

Sixty-nine temporal patterns were found in the study. These patterns were categorized into two groups: i) fetus abnormality (Table 2) complicating pregnancy (Table 3). An example of a mother’s conditions leading to fetus abnormality is 648.01 (Diabetes mellitus) → 655.83 (suspected fetal abnormality), which implies that a mother with antepartum diabetes tends to have an abnormal fetus. An example of a mother’s health conditions leading to complicating pregnancy is 649.73 → 644.03, which indicates cervical shortening increases the risk of threatened premature labor.

Temporal patterns were further categorized into a set of subtypes including obesity, type 2 diabetes, hypertension, opioid dependence, tobacco use, abdominal pain, advanced maternal age, urinary tract infection, hypothyroidism and historical delivery condition such as previous cesarean section. Drug addictions lead to complicating pregnancy. In addition, mothers with drug addictions usually suffer from posttraumatic stress disorder (309.81), viral diseases (647.63) and mental disorders (648.43).

Obesity, diabetes mellitus, and hypertension are correlated with each other (642.03 (Benign essential hypertension) → 648.03 (diabetes mellitus) and 642.23 (transient hypertension) → 649.13 (obesity complicating pregnancy)), and they together complicate pregnancy. Abdominal pain (789.09), advanced maternal age (659.63), urinary tract infection (599.0), hypothyroidism (244.9) and historical cesarean section (654.23) also complicate pregnancy.

### Temporal patterns related to SMM

**Figure 4.**
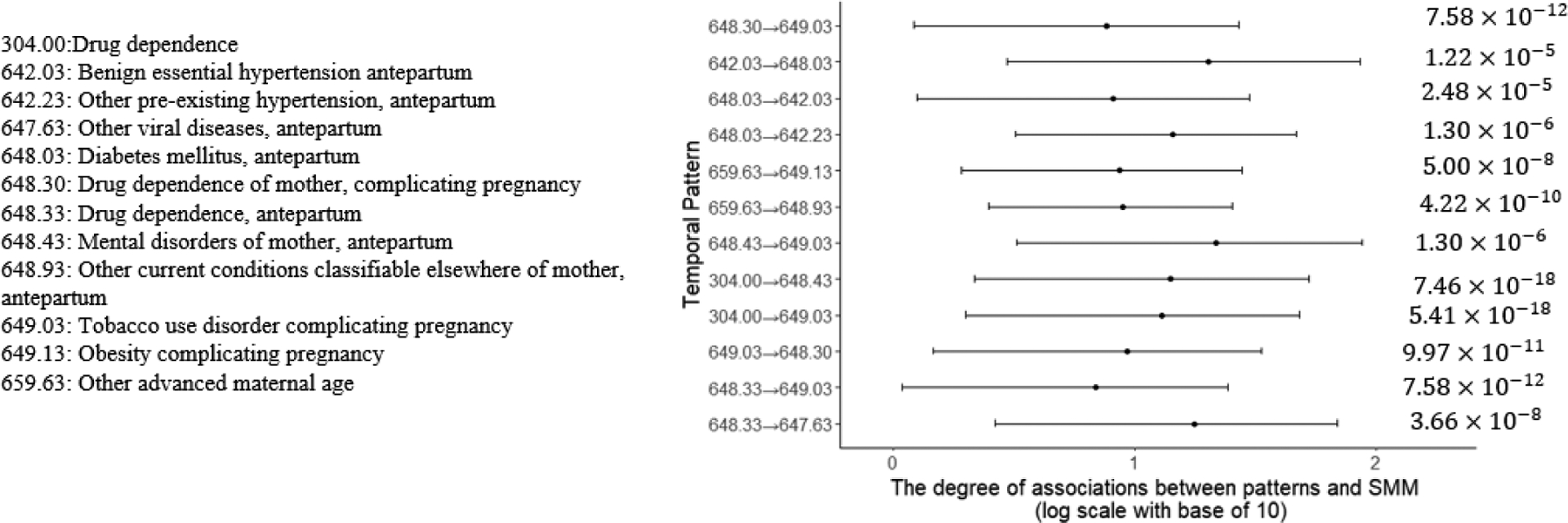
The adjusted relative risk of relationships between temporal patterns and SMM.

Among the 69 temporal patterns, 12 (or 17.4%) were significantly associated with SMM. The degrees of the associations between the 12 patterns and SMM are depicted in Figure 4. For example, women that had antepartum drug dependence and mental disorder had greater than 8-fold risk of SMM in their delivery. Similarly, antepartum conditions hypertension and diabetes (642.03 → 648.03, 648.03 → 642.03 and 648.03 → 642.23) increase the risk of SMM. Other temporal patterns that may lead to SMM include obesity, advanced maternal age, viral disease, and tobacco use disorder.

## Discussion

Severe maternal morbidities (SMM) during delivery often results in adverse outcomes, including a prolonged length of stay and an increase of postpartum readmissions. In this study, we introduced a data-driven framework to infer temporal relational patterns between diagnoses during the antepartum period and analyzed their associations with SMM. This work has two notable findings.

First, we demonstrated that there are temporal patterns that suggest progression paths for SMM. For instance, pre-existing health conditions including hypertension, diabetes mellitus, and obesity appeared to develop into comorbidities in the antepartum period, complicating pregnancy. Additionally, lifestyle factors, such as drug/opioid-dependence and tobacco use disorder, may also complicate pregnancy. It was also found that the well-being of a fetus is associated with adverse antepartum conditions. For instance, mothers with diabetes mellitus tend to have abnormal fetus.

Second, we found that antepartum drug dependence and mental disorder are strongly related to SMM. Early identification of these high-risk mothers and adopt appropriate intervention strategies may improve care quality of SMM management and reduce harms caused by SMM.

At the same time, we note that this is a pilot study, and there are several limitations that need to be addressed.

First, we investigated a small number of SMM instances, which may limit our findings in this study. For instance, many potential temporal patterns may be missed in our investigated patient population. In addition, we only considered diagnoses within antepartum periods, which may miss risk factors of SMM ahead of the antepartum period.

Second, we only studied temporal patterns between diagnosis codes and neglected other rich health information including medications, vital signs, and labs which could provide insights into SMM.

Third, this study was conducted in a single institute and should be expanded in many other institutes to conclude more general recommendations about managing SMM.

Fourth, we used a simple and natural temporal relation learning algorithm to learn temporal patterns. More advanced temporal pattern mining algorithms including Allen’s algebra and graph neural networks can be applied in future studies.

## Conclusion

This research broadens our current knowledge of the continuum of maternal health in the United States by inferring the association between antepartum comorbidities and SMM. Our work suggests that mothers with drug dependence, hypertension, diabetes mellitus, viral disease, and tobacco use disorder have an increased risk of SMM. While further investigation is needed, we believe that healthcare organizations should focus their attention on childbearing women with these conditions in the antepartum period.

## Figures and Tables

**Table 1.** Statistics of age, race, and length of EHR for patients with and without SMM

Factor	SMM *N* = 69	Non-SMM *N* = 6,115
**Age (mean ± SD)**	29.1 ± 5.3	28.9 ± 5.5
**Race (%)**		
**White**	43 (62.3%)	4,129 (67.5%)
**Black**	16 (23.2%)	1,020 (16.7%)
**Asian**	7 (10.2%)	393 (6.4%)
**Other**	3 (4.3%)	573 (9.4%)
**Length of EHR (in weeks)**	78.2 ± 73.1	73.9 ± 68.8

**Table 2.** Temporal patterns related to the fetal abnormality

Subcategories	Temporal Patterns	Summary of the Pattern
**Diabetes mellitus**	648.01 → 655.83	Antepartum diabetes may lead to suspected fetal abnormality
**Cervical shortening**	649.73 → 644.03	Cervical shortening increases the risk of threatened premature labor
**Fetal growth retardation**	764.90 → 656.53	Fetal growth retardation may affect the management of mother
**Uterine size date discrepancy**	649.63 → 656.53	Uterine size date discrepancy is associated with poor fetal growth
**Fetal abnormality**	656 → 655.81	Suspected fetal abnormality and placental problem
**Advanced maternal age**	659.61 → 660	Women with advanced maternal age tend to have obstructed labor
**Antepartum complication**	644.03 → 655.73, 648.93 → 644.03	The antepartum complication may lead to threatened premature labor that is associated with decreased fetal movements

**Table 3.** Temporal patterns related to complicating pregnancy

Subcategories	Temporal patterns	Summary of the Pattern
**Obesity**	278.01 → 649.11, 278.01 → 649.13, 648.01 → 649.11, 278.00 → 649.13, 649.12 → 648.83, 642.93 → 649.13, 659.63 → 649.13, 642.23 → 649.13, 642.03 → 649.13, 649.13 → 642.03	Obesity, especially morbid obesity, complicating pregnancy
**Hypertension**	796.2 → 642.23, 796.2 → 649.13, 401.1 → 642.03, 401.9 → 642.03, 648.03 → 642.03, 642.03 → 649.13, 642.23 → 649.13, 250.00 → 642.23, 648.03 → 642.23, 796.2 → 642.33, 796.2 → 649.13	Pre-existing hypertension, elevated blood pressure reading, antepartum hypertension complicating pregnancy
**Diabetes Mellitus**	250.01 → 648.01, 250.00 → 648.01, 250.00 → 648.83, 250.00 → 642.23, 250.00 → 648.03, 648.01 → 649.11, 790.29 → 648.83, 642.03 → 648.03, 648.03 → 642.23, 648.01 → 649.11	Type I and II diabetes with abnormal glucose tolerance complicating pregnancy; antepartum diabetes may cause some suspected fetal abnormality
**Drug/opioid Dependence**	304 → 648.31, 304 → 648.30, 304.00→ 648.30, 304.00 → 648.33, 304.00 → 648.43, 304.00 → 649.03, 304.01 → 648.31, 304.01 → 648.30, 304.01 → 648.33, 648.31 → 649.01, 648.33 → 647.63	Drug dependence and opioid dependence are related, together complicating pregnancy; drug/opioid dependence is associated with mental disorder
**Tobacco**	648.31 → 649.01, 305.1 → 648.30, 649.03 → 648.30, 304 → 649.03, 648.33 → 649.03	Tobacco use disorder is associated with drug/opioid dependence; both could complicate pregnancy
**Other**	309.81 → 648.30	Other conditions such as posttraumatic stress disorder, thyroid dysfunction, urine infection complicating pregnancy, pain
	244.9 → 648.13, 244.9 → 648.91	
	599.0 → 646.63	
